# Vitamin D Status and the Relationship with Bone Fragility Fractures in HIV-Infected Patients: A Case Control Study

**DOI:** 10.3390/ijms19010119

**Published:** 2018-01-02

**Authors:** Marco Atteritano, Luigi Mirarchi, Emmanuele Venanzi-Rullo, Domenico Santoro, Chiara Iaria, Antonino Catalano, Antonino Lasco, Vincenzo Arcoraci, Alberto Lo Gullo, Alessandra Bitto, Francesco Squadrito, Antonio Cascio

**Affiliations:** 1 Department of Clinical and Experimental Medicine, University of Messina, 98125 Messina, Italy; mirarchi.luigi@tiscali.it (L.M.); evenanzirullo@gmail.com (E.V.-R.); santisi@hotmail.com (D.S.); catalanoa@unime.it (A.C.); alasco@unime.it (A.L.); varcoraci@unime.it (V.A.); albertologullo@virgilio.it (A.L.G.); abitto@unime.it (A.B.); fsquadrito@unime.it (F.S.); 2 Infectious Diseases Unit-ARNAS Civico, Di Cristina, Benfratelli, 90127 Palermo, Italy; iaria.chiara@gmail.com; 3 Department of Health Promotion Sciences and Mother and Child Care “G. D’Alessandro”, University of Palermo, 90127 Palermo, Italy; Antonio.cascio03@unipa.it

**Keywords:** bone ultrasound, HIV, osteoporosis, vertebral fractures, vitamin D

## Abstract

HIV-infected patients show high risk of fracture. The aims of our study were to determine the prevalence of vertebral fractures (VFs) and their associations with vitamin D in HIV patients. 100 patients with HIV infection and 100 healthy age- and sex-matched controls were studied. Bone mineral density was measured by quantitative ultrasound at the non-dominant heel. Serum osteocalcin and C-terminal telopeptide of collagen type 1 served as bone turnover markers. Bone ultrasound measurements were significantly lower in patients compared with controls (Stiffness Index (SI): 80.58 ± 19.95% vs. 93.80 ± 7.10%, respectively, *p* < 0.001). VFs were found in 16 patients and in 2 controls. HIV patients with vertebral fractures showed lower stiffness index (SI) (70.75 ± 10.63 vs. 83.36 ± 16.19, respectively, *p* = 0.045) and lower vitamin D levels (16.20 ± 5.62 vs. 28.14 ± 11.94, respectively, *p* < 0.02). The majority of VFs (87.5%) were observed in HIV-infected patients with vitamin D insufficiency, and regression analysis showed that vitamin D insufficiency was significantly associated with vertebral fractures (OR 9.15; 95% CI 0.18–0.52, *p* < 0.04). VFs and are a frequent occurrence in HIV-infected patients and may be associated with vitamin D insufficiency.

## 1. Introduction

Low bone mineral density (BMD) is a common finding in HIV-infected patients [1,2], and a higher prevalence of osteopenia and osteoporosis up to 70% and 30%, respectively, was reported [3,4]. A systematic review concluded that the probability of osteopenia and osteoporosis could be over 6-fold and almost 4-fold higher in HIV-infected than in the non-infected population, respectively [5], and low BMD is associated with increased risk of bone fragility fractures. HIV-infected patients have been reported to show a higher rate of fragility fractures with an almost 9-fold increased risk of hip fracture and 3-fold increased risk of overall fractures [6,7,8]. Multiple factors appear to be involved in the pathogenesis of bone loss in HIV-infected patients, including HIV viral protein, antiretroviral therapy (ART) side effects, inflammatory cytokines, and bone turnover in addition to traditional risk factors [9,10,11,12,13,14,15]. As is known, vitamin D plays a prominent role in bone and mineral metabolism, and it has been suggested to be involved in the pathogenesis of decreased bone mass and fractures in the general population [16]; thus, it is plausible that vitamin D may exert a role in bone loss and fragility fractures also in HIV-infected patients. The aims of our study were to determine the prevalence of vertebral fractures and to assess the role of bone ultrasound parameters, bone turnover markers, and vitamin D in relation to vertebral fractures in HIV-infected patients.

## 2. Results

**Figure 1:**
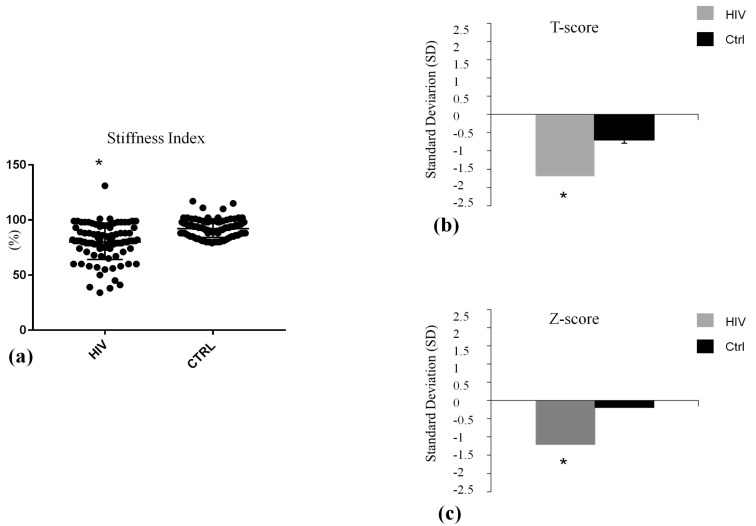
Bone Ultrasound parameters and related T and Z-score in HIV infected (HIV) and control group (Ctrl). * *p* < 0.001. Data are expressed as means and SD. (**a**) The baseline differences of stuffness index between HIV group (HIV) and control group (Ctrl) at 0, was statistically significant; (**b**) The baseline differences of T-score between HIV group and control group (Ctrl) at 0, was statistically significant; (**c**) The baseline differences of Z-score between HIV group and control group (Ctrl) at 0, was statistically significant.

**Figure 2:**
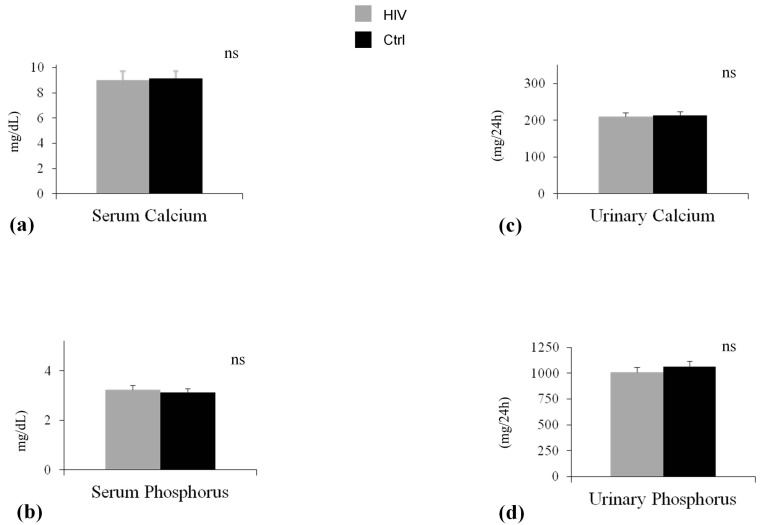
Serum and 24 hours urinary escretion of Calcium and Phosphorus in two groups. Data are expressed as means and SD. (**a**) The baseline differences of Serum Calcium between HIV group (HIV) and control group (Ctrl) was not statistically significant (*p* = 0.13); (**b**) The baseline differences of serum Phosphrous between HIV group (HIV) and control group (Ctrl) was not statistically significant (*p* = 0.67); (**c**) The baseline differences of urinary Calcium between HIV group (HIV) and control group (Ctrl) was not statistically significant (*p* = 0.82); (**d**) The baseline differences of urinary Phosphrous between HIV group (HIV) and control group (Ctrl) was not statistically significant (*p* = 0.31).

**Figure 3:**
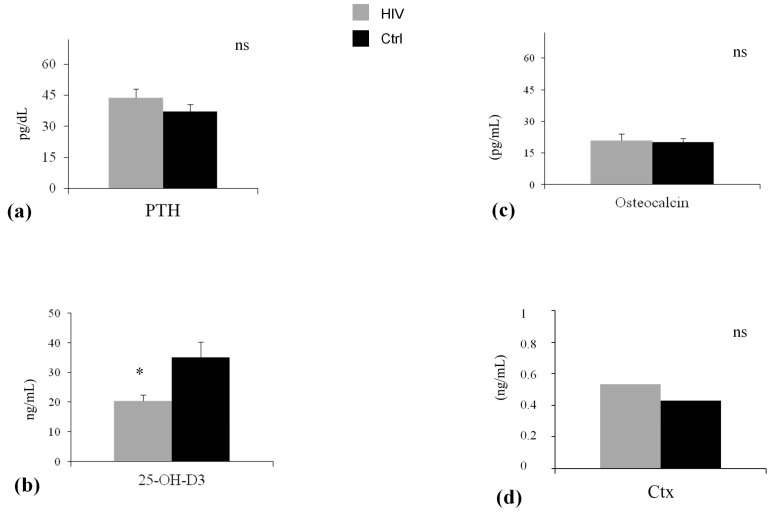
Serum parathyroid hormone (PTH), 25(OH)D3 and bone turnover markers in two groups. * *p* < 0.001. Data are expressed as means and SD. (**a**) The baseline differences of serum parathyroid hormone between HIV group (HIV) and control group (Ctrl) was not statistically significant (*p* = 0.08); (**b**) The baseline differences of Serum 25-OH-D3 between HIV group (HIV) and control group (Ctrl) was not statistically significant (*p* = 0.0001); (**c**) The baseline differences of osteocalcin between HIV group (HIV) and control group (Ctrl) was not statistically significant (*p* = 0.13); (**d**) The baseline differences of CTx between HIV group (HIV) and control group (Ctrl) was not statistically significant (*p* = 0.26).

Demographic and clinical features of participants are shown in Table 1. As expected in light of the matching design of the study, chronological age (*p* = 0.73) and sex (*p* = 1.00) and family history of osteoporosis and fracture (*p* = 0.79) were comparable in HIV-infected patients and controls. Other relevant factors associated with BMD were also non-significantly different in the two groups (Table 1). The ultrasound parameter at calcaneus, SI was significantly lower in HIV-infected patients in comparison with control (SI: 80.58 ± 19.95% vs. 93.80 ± 7.10%, *p* < 0.001) (Figure 1a). Serum levels of calcium and phosphorus, urinary calcium and phosphorus (Figure 2a–d), and bone turnover markers (Figure 3c,d) did not differ significantly between the study groups. The level of PTH was higher, albeit not significantly, in patients compared with control subjects (Figure 3a). 25-Hydroxivitamin D3 (25(OH)D3) level was significant lower (20.29 ± 4.05 ng/mL vs. 35.77 ± 6.50 ng/mL, *p* < 0.001) in HIV-infected patients compared with controls (Figure 3b). Lateral spine X-ray documented single or multiple VFs in 16 patients (16%) and 2 controls, with a significantly different prevalence in the two groups (*p* < 0.05). Ten patients had a single Grade-1 fracture, 5 patients had two Grade-3 fractures, and 1 patient had multiple fractures (two Grade-1 and three Grade-3). In HIV-infected patients, the comparison between subjects without vertebral fractures (No-VFs) and subjects with VFs showed that this latter group reported a poorer measurement of SI (83.36 ± 16.19 vs. 70.75 ± 10.63; *p* = 0.045) (Table 2) and a lower 25(OH)D3 (28.14 ± 11.94 vs. 16.2 ± 5.62; *p* < 0.02) (Table 2). No statistically significant differences were found in age, BMI, smoking habits, and viro-immunological markers between fractured and non-fractured patients.

VFs were significantly associated with the insufficiency of 25(OH)D3, and these associations were significant after adjusting for age, renal function, and systemic hypertension. No other significant associations were found between 25(OH)D3 serum levels and the other clinical parameters assessed in HIV-infected patients.

## 3. Discussion

Our data show that HIV-infected patients exhibited lower BMD as assessed by bone quantitative ultrasound at the heel. SI measurements and the related T- and Z-score values were significantly lower in the HIV-infected patients compared with the control group, the latter being matched with patients not only for sex and age but also for other known major risk factors for osteoporosis and fractures.

The X-ray of the thoracic and lumbar spine showed single or multiple VFs, according to the Genant semiquantitative classification of VFs, in 16 HIV-infected patients with a prevalence of 16% and in only two subjects from the control group. These data are consistent with the current literature and highlight the high risk of fracture in this cohort [17,18,19]. Age, chronic renal insufficiency, and steroid use were previously associated with increased risk of fractures in HIV-infected patients [20,21]. However, since there are no significant differences between our HIV-infected patients and the controls, when considering these variables, it is reasonable to rule out that a low BMD and VFs are related to these factors.

Another important determinant of bone loss and fragility fractures in HIV-infected patients is represented by ART, in fact several studies, but not all, have proved the association between ART exposure and osteoporosis and/or fractures [22,23,24].

In our study, 94% of HIV-infected patients were treated with ART, so it was not possible to analyze the impact of the therapy, per se, on BMD and fractures. Interestingly, the HIV-infected patients with VFs reported a lower value of SI in comparison with HIV-infected patients without VFs; this result is consistent with previous data on the role of quantitative ultrasound in the detection of bone fragility. Quantitative ultrasound is able to reflect different physical properties of bone (e.g., the density, elasticity, and microarchitecture) and provide information on fracture risk [25,26,27,28,29,30].

Our data may indicate that bone ultrasound at the non-dominant heel may be used as a reliable tool to test bone health in HIV-infected patients.

Great attention has been given to the field of vitamin D in HIV-infected patients over the last few years, and epidemiological data suggest that HIV-infected patients, also from different geographical locations, including Europe, American, and Australia have a high prevalence of vitamin D insufficiency [31,32,33]. In our cohort, the prevalence of insufficiency of vitamin D3 in HIV-infected patients was 50%, significantly higher compared to the control group (20%). Diet and exposure to the sun are the main factors that regulate vitamin D3 levels and its activity in each subject [34]. The average self-reported sun exposure was the same in both groups, so sun exposure is not likely to be the cause of the low 25(OH)D3 level showed in HIV group than controls. Poor vitamin D status is a well-established risk factor for bone disease in the general population [35]. In addition, recent data suggest that vitamin D may exert non-skeletal functions, having a role in cardiovascular and immune regulation [36,37,38]. Moreover, in HIV human macrophages exposed to mycobacterium tuberculosis, vitamin D pretreatment restored critical responses, supporting a potential role for exogenous vitamin D as a therapeutic adjuvant in *M. tuberculosis* infection in HIV(+) persons [39]. The extent to which vitamin D deficiency contributes to bone loss and enhance fracture risk in the HIV-infected patients is largely unknown. Our study demonstrated that vitamin D insufficiency is associated with prevalent vertebral fractures in HIV-infected patients: 87.5% of HIV-infected patients presenting VFs had serum with a vitamin D value lower than 30 ng/mL, and vitamin D was predictive of fractures according to regression analysis. The finding that insufficient 25-hydroxyvitamin D3 levels (<30 ng/mL) are associated with VFs support the clinical relevance of vitamin D preserving bone health also in HIV-infected patients, and supports the inclusion of this modifiable risk factor as part of the screening for bone fragility in HIV-infected patients.

We acknowledge that our study has some limitations, including the small sample size and its cross-sectional and observational nature, that need to be considered when interpreting the final results. Another limitation was not to compare the population of HIV with another chronic disease to better understand the impact and specificity of HIV on bone loss and vertebral fragility fractures.

## 4. Materials and Methods

### 4.1. Subjects

The study was approved by the Local Ethics Committee for Medical Research, Messina University Hospital “G.Martino” and carried out in accordance with the Helsinki Declaration. All subjects gave their informed written consent to enter the study. One hundred consecutive patients with diagnosis of HIV infection referred from July 2012 to February 2015 to the Infection Unit of the University Hospital of Messina were evaluated. Eligibility criteria required the absence of clinical or laboratory abnormalities that suggested cardiovascular, hepatic, or renal disorders; coagulopathy, the use of oral or transdermal estrogen, progestin, androgen, or other steroids; previous or current use of bone active agents (e.g., biphosphonates), the use of cholesterol-lowering therapy, cardiovascular medications, or any other therapy that could influence bone metabolism, in particular, systemic or local corticosteroids. Of the 141 patients evaluated, 100 fulfill the inclusion criteria and were included in this cross-sectional study. One hundred healthy volunteers matched for sex and age served as the control group. All participants in both groups were white subjects from South Italy. Clinical data, including smoking status (current, former, never), physical activity, food energy, calcium intake, and sun exposure, were obtained by interview, and weight and height were determined during physical examination. The nutritional variables were determined using 24 h recall. None of the subjects in either group were on supplementation with calcium and/or vitamin D.

### 4.2. Bone Ultrasound Parameters and Vertebral Fractures Assessment

All subjects underwent a BMD evaluation by quantitative ultrasound (QUS) at the non-dominant heel by Achilles Insight, (Lunar, Madison, WI, USA). The Achilles Plus measures the speed of sound (SOS), broadband ultrasound attenuation (BUA), and a clinical index called stiffness index. Stiffness index (SI) is calculated automatically by the software according to the following formula: SI = (0.67 × BUA + 0.28 × SOS) − 420. Standardized procedures were carried out for patient positioning, data acquisition, and system calibrations. The manufacturer’s phantom was used for system calibrations. The coefficient of variation for SI was 1.7%.

All participants also underwent a lateral thoracic and lumbar spine X-ray to ascertain the presence of VFs. All radiographs were assessed by two blinded physicians separately according to the quantitative methods (quantitative vertebral morphometry, QVM) using a dedicated software (MorphoXpress). Anterior, middle, and posterior heights of vertebral T4 to L4 were measured. According to the Genant classification, a vertebral fracture was defined based on reduction in anterior, middle, and/or posterior height: Grade 1: 20–25% reduction; Grade 2: 25–40% reduction; Grade 3: >40% reduction [40].

### 4.3. Biochemical Data

Blood samples were obtained by antecubital venipuncture between 8 a.m. and 11 a.m. after an overnight fast and a 10 min rest. The first 4–5 mL of blood were not used. Blood was collected in refrigerated vacutainers containing an anticoagulant mixture provided by Booehringer-Mannheim, immediately placed on ice, and centrifuged, within a few minutes, at 2000× *g* for 20 min at 4 °C, and the plasma was frozen at −80 °C until assayed. Osteocalcin (BGP) was performed using an immunoenzymatic assay (Pantec, Turin, Italy). Serum levels of C-telopeptide of type I collagen (CTx) was assessed using the Elecys 2010 Immunoassay System (Roche, Basel, Switzerland). Serum calcium, serum phosphorus, and urinary creatinine were measured by automated routine procedures. Parathyroid hormone (PTH) and 25-hydroxyvitamin D3 were measured using high-performance liquid chromatography (Bio-Rad Laboratories S.r.l., Segrate, Milan, Italy). Vitamin D deficiency was defined as plasma levels of 25(OH)D3 <20 ng/mL, insufficiency between 20 and 30 ng/m, and normal >30 ng/mL. The intra- and inter-assay CV were <10% for both tests. The intra- and inter-assay CV were <10% for both tests.

### 4.4. Statistical Analysis

Statistical analyses were performed using Statistica 8 (Statsoft, Inc., Tulsa, OK, USA). Values are expressed as mean SD or percentage. Comparisons between groups were performed by with a Student’s *t*-test. The percentage of each variable was compared between groups by Fisher’s exact test. A chi-squared test (χ^2^) with Yates correction was conducted to assess the individual association between independent variables and the presence of insufficiency 25(OH)D3. Multivariate logistic regression analysis was used to adjust for confounders. Values of *p* <0.05 were considered statistically significant.

## 5. Conclusions

In conclusion, our data showed a high prevalence of VFs in HIV-infected patients and highlight the role of insufficiency of vitamin D3 in increasing the risk of VFs.

## Figures and Tables

**Table 1:** Anthropometric data and risk factors for fractures in two groups; results are expressed as number, means ± S.D., and percentage.

Characteristics	HIV (*n* = 100)	Controls (*n* = 100)	*p*
Age (years)	45.36 ± 12.02	44.85 ± 9.30	0.73
Body Mass Index (kg/m^2^)	25.01 ± 4.23	24.80 ± 4.65	0.73
Sex (F/M)	84/16	84/16	1.00
Duration of disease (years)	16.29 ± 6.48	0	N/A
Current tobacco use, *n* (%)	22 (22)	20 (20)	0.88
Alcohol intake (mL/week)	450 ± 100	450 ± 80	1.00
Calcium intake (mg/day)	510 ± 117.88	520 ± 122.53	0.55
Food energy (Kcal/day)	1350 ± 200	1360 ± 150	0.23
Low physical activity	70	68	0.66
Supplementation with calcium, *n*	0	0	N/A
Supplementation with vitamin D, *n*	0	0	N/A
Current steroid use, *n* (%)	0	0	N/A
Antiretroviral therapy exposure			
Naive, *n* (%)	6 (6)	0	N/A
Experienced, *n* (%)	94 (94)	0	N/A
Sunlight exposure >5 h/week, *n* (%)	26 (26)	26 (26)	0.52
Family history of osteoporosis			
and/or fractures, *n* (%)	12 (12)	14 (14)	0.79
Fall history, *n* (%)	15 (15)	17 (17)	0.87

N/A: not applicable.

**Table 2:** Bone ultrasound parameter, laboratory data, and vertebral fractures in HIV infection and control group; results are expressed as number, means ± S.D. and percentage.

Parameter	HIV (*n* = 100)	Control Group (*n* = 100)
Vertebral fractures (VFs), *n* (%)	16 (16) *	2 (2)
Stiffness index (SI) (%)	80.58 ± 19.95 ^§^	92.18 ± 8.06
T-Score (D.S.)	−1.70 ± 0.50 ^§^	-0.70 ± 0.30
Z-Score (D.S.)	−1.20 ± 0.40 ^§^	-0.10 ± 0.30
Osteocalcin (pg/mL)	21.31 ± 4.95	20.33 ± 4.35
C-terminal telopeptide (ng/mL)	0.53 ± 0.28	0.48 ± 0.35
Parathyroid hormone level (pg/dL)	43.72 ± 25.40	37.64 ± 23.60
25-hydroxivitamin D3 (ng/mL)	20.29 ± 4.05 ^§^	35.77 ± 6.50
Calcium (mg/dL)	9.02 ± 0.47	9.12 ± 0.46
Phophate (mg/dL)	3.11 ± 0.57	3.14 ± 0.43
Urinary calcium (mg/24 h)	210.29 ± 84.05	213.93 ± 140.35
Urinary phophate (mg/24 h)	1010.29 ± 260.05	1063.53 ± 461.50
Vitamin A (g/dL)	62.65 ± 13.89	62.33 ± 13.22
Creatinine (mg/dL)	0.88 ± 0.07	0.89 ± 0.09

* *p* < 0.05; ^§^ *p* < 0.001.
